# Commentary: Exogenous Testosterone Rapidly Increases Aggressive Behavior in Dominant and Impulsive Men

**DOI:** 10.3389/fpubh.2016.00229

**Published:** 2016-10-12

**Authors:** Leo Sher

**Affiliations:** ^1^James J. Peters Veterans’ Administration Medical Center, Bronx, NY, USA; ^2^Icahn School of Medicine at Mount Sinai, New York, NY, USA

**Keywords:** men, testosterone, aggression, dominance, suicide

I read with interest a research report, “Exogenous testosterone rapidly increases aggressive behavior in dominant and impulsive men” by Carré et al. that was recently published in *Biological Psychiatry* (1). Healthy adult men were administered either testosterone or placebo, and then engaged in a decision-making game that assesses aggressive behavior in response to social provocation. The researchers also examined the extent to which testosterone influence on aggressive behavior depends on variability in trait dominance and/or trait self-control. The authors observed that exogenous testosterone on its own did not modulate aggressive behavior. However, they found that testosterone can quickly (within 60 min) potentiate aggressive behavior among men with dominant or impulsive personality styles. More specifically, testosterone increased aggressive behavior, but only among men scoring high in trait dominance or men scoring low in trait self-control. There was no effect of testosterone on aggressive behavior in men scoring low in trait dominance or high in trait self-control.

The observation by Carré et al. (1) is consistent with our case report published in 2013 (2). We reported a case of a middle-age man with a history of bipolar disorder with psychotic features, substance use disorder, and aggressive behavior who became violent some hours after receiving a testosterone injection and hit his wife in the abdominal area which led to an internal bleeding and resulted in her death. He admitted to the police to pushing his wife down on the ground and kicking her in the stomach. It is difficult to establish a clear relation between the testosterone administration and the murder in this case. However, the testosterone administration possibly contributed to homicide.

The observation by Carré et al. (1) may shed some light on the relationships between testosterone and suicidal behavior. Several studies suggested that testosterone may be involved in the pathophysiology of suicidal behavior (3–7) while at least two studies did not support this assertion (8, 9). It has been proposed that there are substantial similarities between aggression against the self and aggression against others, based on the clinical and epidemiological findings that some suicide attempters may share personality traits with violent criminals (10). An association between aggression and suicidal behavior has been observed by multiple researchers (11–15). For example, it has been shown that high aggression predicts suicidal acts (11). We have also shown that the higher prevalence of suicide attempters among depressed patients with a history of alcoholism compared to depressed patients without a history of alcoholism was related to higher aggression scores in the group with alcoholism (12). Thus, men who become impulsive–aggressive when the testosterone levels are higher may also have a tendency to commit a suicidal act when the testosterone concentrations are higher, i.e., these men may be potential suicide attempters. Identification of such men may help to prevent suicide attempts. This may be an interesting and important new avenue in suicide prevention work.

## Author Contributions

The author confirms being the sole contributor of this work and approved it for publication.

## Conflict of Interest Statement

The author declares that the research was conducted in the absence of any commercial or financial relationships that could be construed as a potential conflict of interest.
